# Alone but not lonely: The relationship between COVID-19 social factors, loneliness, depression, and suicidal ideation

**DOI:** 10.1371/journal.pone.0261867

**Published:** 2021-12-23

**Authors:** Ana Rabasco, Vincent Corcoran, Margaret Andover

**Affiliations:** Department of Psychology, Fordham University, Bronx, New York, United States of America; Konkuk University, REPUBLIC OF KOREA

## Abstract

**Objective:**

Since the start of the COVID-19 pandemic, there have been concerns that social distancing may negatively impact mental health, particularly with regards to loneliness, depressive symptoms, and suicidality. The current study explored how aspects of social distancing, communication, and online support from October 2020 to December 2020 related to loneliness, depressive symptoms, and suicidal ideation.

**Method:**

Participants (*n* = 216) who self-identified as having mental health diagnoses were recruited and completed questionnaires online.

**Results:**

Findings showed that COVID-19 related social contact, particularly electronic social contact, is associated with decreased loneliness, suicidal ideation, and depression. Online emotional support was significantly associated with decreased loneliness and depressive symptoms. Social distancing practices were not associated with increased loneliness, suicidal ideation, and depression.

**Conclusions:**

Our findings underscore the importance of leveraging electronic methods of social connection, especially among individuals who are at risk for suicide or depression.

## Introduction

The first case of the novel coronavirus (SARS-CoV-2), the cause of coronavirus disease 2019 (COVID-19), was identified in the United States in January 2020. In March 2020, the World Health Organization (WHO) declared COVID-19 a global pandemic, and efforts to contain the spread of the virus were widely instituted [1]. These efforts included social distancing, or the practice of encouraging individuals to maintain a six-foot distance from others who are not members of their household, both indoors and outdoors, to reduce the rate of droplet transmission of pathogens [2]. While social distancing is considered a necessary and essential measure to reduce the incidence of COVID-19, it could impact mental health [3, 4], including increased loneliness, depressive symptoms, and suicidality [5].

Recent research has indicated that COVID-19 social distancing restrictions are associated with increased social isolation and loneliness among adults [5] and older adults [6]. Social isolation is characterized by having minimal social contacts and deficits in quality relationships [7], while loneliness involves the affective and cognitive discomfort that arises from perceiving oneself as being alone [8]. This increase in social isolation and loneliness is a concern, as a significant body of research has found these factors to be associated with increased risk for depression and suicidality [9, 10]. Theories of suicide, including Joiner’s (2005) interpersonal psychological theory of suicide (IPTS) and Klonsky and May’s (2015) three-step theory (3ST), identify a lack of social belonging and connectedness as key risk factors for suicidality [11, 12].

Depressive symptoms and suicidal ideation (SI) have been observed to be at increased levels during the COVID-19 pandemic compared to data collected pre-pandemic during the same time period [13]. Previous research conducted during March and April 2020, a time of intense implementation of public health measures to contain COVID-19, found that social distancing was associated with psychological distress, including depression [14]. Additionally, perceived impact of social distancing public health policies (both on social/work routines and on mental health) has been found to be associated with suicide attempts in the past month [15]. This is consistent with research on suicidality during emerging viral disease outbreaks prior to COVID-19, as slight but significant increases in deaths by suicide during those times have been noted [16].

Forms of electronic communication may help to mitigate some of the negative effects of social distancing. Currently, internet use in the United States approaches near universal levels, with 93% of adults reportedly accessing online spaces, and 72% use at least one social media site [17, 18]. The importance of online technology has been highlighted by the current pandemic, as 53% of individuals reported the internet has been “essential to them personally” during the ongoing COVID-19 global health crisis [19]. Internet use during the pandemic extends beyond work and education related functions, as many indicate they have used the internet in novel ways to stay socially connected. For example, 32% of adults reported having attended a social gathering or party online, 20% used the internet to livestream a concert or play, and 18% reported participating in an online fitness class/workout [19].

The prevalence of novel internet use to increase social connection during the COVID-19 pandemic demonstrates the ever-increasing need to better understand how individuals receive support through the internet, and whether online social interaction can mitigate the loneliness, depressive symptoms, and suicidality associated with social distancing. Social support, or emotional or pragmatic help received from interpersonal relationships [8], has been shown to both promote well-being and buffer against negative health outcomes [20], including suicidality [21]. Research conducted prior to the COVID-19 pandemic comparing in-person to online social support found that while in-person social support had stronger effects on self-esteem and depressive symptoms, online social support still had a significant protective effect [22]. The COVID-19 health crisis has highlighted the importance of online social support, and researchers have called for a better understanding of how access to internet technology may buffer the experience of loneliness [4].

Since the start of the COVID-19 pandemic, there have been concerns that social distancing may negatively impact mental health, particularly with regards to loneliness, depressive symptoms, and suicidality. However, previous research has yet to examine how electronic communication and online social support, two key facets of people’s social interaction during the COVID-19 pandemic, are associated with loneliness, depressive symptoms, and SI. Therefore, the purpose of the current study was to explore how aspects of social distancing, communication, and online support from October 2020 to December 2020 related to loneliness, depressive symptoms, and SI. Fall 2020 was a phase of COVID-19 marked by a lack of vaccine, increasing infection rates, and restrictions on social gatherings [1].

## Methods

### Participants

The sample consisted of 216 adults recruited through ResearchMatch, an online platform that matches individuals interested in participating in research with researchers actively searching for volunteers [23]. Individuals who had indicated on ResearchMatch that they had mental health diagnoses received recruitment announcements for the present study. Participant inclusion criteria was as follows: 1) ability to read English, and 2) 18 years of age or older. There were no exclusion criteria for the study. Participant characteristics are included in Table 1.

**Table 1:** Sample characteristics (*n* = 216).

	*Mean (SD) or %*	*N*
Age	39.24 (15.77)	----
Gender		
Man	14%	31
Woman	78%	168
Transgender	1%	3
Nonbinary	6%	13
Race		
American Indian or Alaska Native	1%	2
Asian	5%	10
Black or African American	3%	7
White or Caucasian	83%	178
Multiracial	1%	2
Other	5%	10
Ethnicity		
Hispanic or Latinx	6%	12
Non-Hispanic or Latinx	94%	203
Sexual Orientation		
Asexual	4%	8
Bisexual	13%	29
Gay	3%	7
Heterosexual	62%	139
Lesbian	3%	7
Pansexual	4%	9
Queer	3%	7
Questioning/unsure	1%	2
Other	3%	6
Region		
Northeast	19%	38
Southeast	31%	63
Midwest	27%	55
Southwest	3%	6
West	21%	43

### Measures

Participants were asked to report their demographic information (age, gender, race, ethnicity, sexual orientation, state of residence, and education level). Loneliness was assessed using the UCLA Loneliness Scale, a 20-item self-report measure of individual’s subjective feelings of loneliness and social isolation (Cronbach’s alpha = .95) [24]. Depressive symptoms were assessed using the Beck Depression Inventory-II (BDI-II), a 21-item self-report measure used to assess depressive symptoms occurring during the preceding two-week period (Cronbach’s alpha = .91) [25]. SI was measured using the Beck Scale for Suicidal Ideation (BSS), a 21 item-self report measure consisting of 21 sets of statements assessing suicidality, including wish to live, wish to die, reasons for living, and suicide planning behaviors in the past week (Cronbach’s alpha = .94) [26]. Online social support was measured using the second set of items on the Online Social Support Scale, which assesses for four different domains of online social support that include: esteem/emotional support, social companionship, informational support, and instrumental support (Cronbach’s alphas: Esteem/Emotional Support = .97; Social Companionship = .95; Informational Support = .96; Instrumental Support = .90) [22].

Finally, fourteen items were derived from the Johns Hopkins University COVID-19 Community Response Survey to measure social distancing behaviors and social contact since March 1^st^, 2020. The current study examined the following items: 1) duration of social distancing (in days), 2) number of household occupants while social distancing, 3) last time (in days) one had face to face contact with someone outside one’s household (anyone seen in person, even at a 6 ft distance), 4) last time (in days) one had physical contact with another person (e.g., a friendly hug), 5) last time (in days) one left their house for any reason, and 6) number of friends or relatives one has seen or hear from at least once a month. Five questions assessed for the frequency of contact with friends and relatives since March 2020 on a scale from 1 (never) to 4 (always) using the following methods: texting, talking by phone, talking by video, meeting in person at someone’s home, meeting in person at a public place. The overall average frequency of electronic communication score was the mean of the texting, talking by phone, and talking by video items. The overall average frequency of in-person communication score was the mean of the meeting in person at someone’s home and meeting in person at a public place items [27].

### Procedure

First, ResearchMatch registrants who indicated having a history of mental health diagnoses (e.g., depression) were emailed a study announcement by ResearchMatch. Individuals who indicated their interest were emailed a link to the Qualtrics survey. All study procedures occurred online. Participants completed screening questions to confirm the study’s inclusion criteria. Those who met inclusion criteria were asked to indicate informed consent by clicking a button after reading the informed consent form and passing a brief quiz. Participants then completed a series of questionnaires. At the end of the survey, participants were debriefed and offered an option to enter their name into a gift card raffle. Data were collected from October 2020 to December 2020. Fordham University’s Institutional Review Board approved the study protocol.

## Results

Table 2 includes descriptive statistics for study variables. We first examined demographic variables as potential covariates. Ethnicity and sexual orientation were significantly associated with loneliness score and were therefore included as covariates in all analyses with loneliness as the outcome. Ethnicity, age, and sexual orientation were significantly associated with depressive symptoms; therefore, they were included as covariates in all analyses with depressive symptoms as an outcome. Finally, race, age, and sexual orientation were significantly associated with SI and were included as covariates in all analyses with SI as the outcome (see Table 3).

**Table 2:** Descriptive statistics for variables of interest.

	*Mean (SD) or %*	*Range or N*
BDI Score	20.3 (11.42)	0–50
BSS Severity Score	3.60 (6.25)	0–29
Presence of Suicidal Ideation Past Week		
Yes	43%	92
No	57%	123
Lifetime History of Suicide Attempt		
Yes	39%	83
No	61%	132
Number of Days Social Distancing	202.29 (79.94)	0–400
Number of Household Occupants (including self)	2.71 (2.09)	1–20
Number of Days Since Face-to-Face Contact with Non-Household Occupant	5.59 (11.14)	0–80
Number of Days Since Physical Contact with Another Person	32.53 (119.05)	0–1,500
Number of Days Since Leaving House	4.09 (17.61)	0–230
Number of People in Contact with Each Month	7.43 (4.38)	1–17
Frequency of Contact (with those in most contact with)		
Less Than Monthly	4%	8
Monthly	10%	21
Few Times a Month	15%	31
Weekly	16%	35
Few Times a Week	31%	67
Daily	24%	51
OSSS subscales		
Emotional/Esteem	18.41 (10.12)	0–40
Social Companionship	15.67 (9.73)	0–40
Informational	16.27 (9.60)	0–40
Instrumental	7.15 (7.01)	0–30
UCLA-LS	49.97 (13.74)	2–75

*Note*. BDI = Beck Depression Inventory. BSS = Beck Scale for Suicidal Ideation. OSSS = Online Social Support Scale. UCLS-LS = UCLS-Loneliness Scale.

**Table 3:** Associations between outcomes and demographic covariates.

Measure	Loneliness		Depressive Symptoms		Suicidal Ideation	
	*r*		*r*		*r*	
Age	-.10		-.26**		-.29**	
	*F/t*	*h* ^*2*^ */d*	*F/t*	*h* ^*2*^ */d*	*F/t*	*h* ^*2*^ */d*
Race	0.72	0.02	0.75	0.02	2.91*	0.07
Ethnicity	2.01*	0.65	2.92**	0.81	1.82	0.62
Gender	0.13	0.001	0.48	0.01	0.49	0.01
Sexual Orientation	-2.94**	0.43	-3.13**	0.44	-2.81**	0.42

*Note*. Sexual orientation dichotomized to LGBQ+ and heterosexual.

* *p* < .05,

** *p* < .01,

*** *p* < .001.

### Loneliness

A multiple linear regression was conducted with all six COVID-19 social factors as independent variables and total loneliness score as the dependent variable (See Table 4). The number of people in an individual’s household was significantly positively associated with increased loneliness. The number of people an individual was in contact with was significantly negatively associated with loneliness. No other factors (duration of social distancing, last time since face to face contact, last time since physical contact, last time leaving home) were uniquely associated with loneliness. Next, a multiple regression was conducted to determine whether type of social contact, either electronic (average frequency of texting, phone, and video contact) and/or in-person (average frequency of meeting in person at someone’s home or at a public place), was associated with loneliness (See Table 4). Frequency of electronic communication was negatively associated with loneliness, but frequency of in-person communication was not uniquely associated with loneliness. Lastly, a multiple linear regression was conducted with types of online support entered as independent variables and total loneliness score entered as the dependent variable. Only receiving online emotional/esteem support was significantly associated with lower loneliness scores (see Table 4).

**Table 4:** Multiple regression analyses.

Social COVID Variables						
	Loneliness		Depressive Symptoms		Suicidal Ideation	
Predictors	*t*	*β*	*t*	*β*	*t*	*β*
1) Social Distancing Duration	-1.97	-.08	-1.59	-0.11	-0.87	-0.06
2) Household Occupants	2.37*	1.61	1.95	0.14	0.02	0.001
3) Days Since Face-to-Face Contact	0.82	0.06	0.40	0.03	0.49	0.04
4) Days Since Physical Contact	1.64	0.11	1.32	0.09	0.06	0.01
5) Days Since Leaving House	-0.59	-0.04	-0.29	-.02	-0.56	-0.04
6) Number of People in Contact With	-4.55***	-0.31	-3.73***	-0.26	-2.39*	-0.17
Frequency of Electronic vs. Face-to-Face Communication						
	Loneliness		Depressive Symptoms		Suicidal Ideation	
Predictors	*t*	*β*	*t*	*β*	*t*	*β*
1) Electronic Communication	-3.94***	-0.27	-3.25**	-0.21	-3.16**	-0.21
2) Face-to-Face Communication	-0.59	-0.04	-0.15	-0.01	0.48	0.03
Online Social Support						
	Loneliness		Depressive Symptoms		Suicidal Ideation	
Predictors	*t*	*β*	*t*	*β*	*t*	*β*
1) Emotional/Esteem Support	-2.57*	-0.29	-2.43*	-0.28	-1.01	-0.12
2) Social Companionship	-0.23	-0.03	0.29	0.04	-1.01	-0.15
3) Informational Support	0.77	0.09	1.57	0.18	-1.25	0.15
4) Instrumental Support	-1.02	-0.09	-1.39	-0.12	0.10	0.01

### Depressive symptoms

Analyses were repeated with depressive symptoms as the outcome (see Table 4). Only being in contact with a larger number of people was significantly associated with decreased depressive symptoms. No other social factors were uniquely associated with depressive symptoms. Next, a multiple regression was conducted to determine whether type of social contact, either electronic and/or in-person, was associated with depressive symptoms (see Table 4). Frequency of electronic communication was negatively associated with depressive symptoms, but frequency of in-person communication was not uniquely associated. Again, a multiple linear regression was completed with types of online social support entered as independent variables, but with depressive symptoms as the dependent variable. Only online emotional/esteem support was significantly associated with lower depressive symptoms (See Table 4).

### Suicidal ideation

Analyses were repeated a third time with SI as the outcome (See Table 4). Only being in contact with a larger number of people was significantly associated with decreased SI. No other social factors were uniquely associated with SI Then, a multiple regression was conducted to determine whether type of social contact, either electronic and/or in-person, was associated with SI (See Table 4). Frequency of electronic communication was negatively associated with SI, but frequency of in-person communication was not uniquely associated (see Table 4). In a final multiple regression conducted with types of received online social support, no category was associated with SI (See Table 4).

## Discussion

The current study explored the relationships between COVID-19 social factors (social distancing, communication, and online support) and the mental health outcomes of loneliness, depressive symptoms, and SI severity during Fall 2020 of the COVID-19 pandemic. First, we found that being in contact with a larger number of people was associated with significantly lower loneliness, depressive symptoms, and SI severity. This aligns with previous research conducted prior to the COVID-19 pandemic [21]. In addition, living in a home with a greater number of people was associated with increased loneliness, but not depressive symptoms or SI. This highlights that loneliness and social isolation are two separate concepts; an individual can be around others and still feel lonely [28]. It is possible that those participants who lived in larger households felt a lack of emotional connection with those they lived with, especially of they lived with others for financial, rather than social, reasons.

Interestingly, the duration of social distancing was not associated with loneliness, SI, or depressive symptoms. This differs from research conducted on the relationship between social distancing and suicide during the 1918 Spanish Flu pandemic, which found that social distancing was associated with increased suicide death rates [29]. Today, electronic social contact could be a key protective factor in the relationship between social distancing and suicidality and depression. In addition, we specifically recruited individuals who had indicated past or current mental health symptoms. Hamza et al. (2021) found that individuals with preexisting mental health concerns showed improving or similar mental health from May 2019 to May 2020, while individuals without preexisting mental health concerns showed declining mental health during that time frame [30]. They also found that loneliness levels remained the same for individuals without preexisting mental health concerns but increased for individuals without preexisting mental health concerns; authors suggested that increasing loneliness may in part account for increasing distress. It is possible that if a sample without mental health concerns had been included in this study, social distancing may have been associated with increased loneliness, SI, or depressive symptoms.

We then examined how both in-person social contact and electronic contact were related to loneliness, depressive symptoms, and SI. Electronic contact significantly statistically predicted lower depressive symptoms, loneliness, and SI severity levels, while in-person social contact did not. Some research conducted since the onset of COVID-19 has identified online social connection as similarly protective against depression [31]. However, other recent research conducted by Rosenberg et al. (2021) has identified that only frequent in-person social connections were associated with lower levels of depression, while electronic social connections were not [31]. These contradictory findings may be due to the time period in which the data was collected. Rosenberg et al. (2021) collected their data in April 2020, while the data from this project was collected from October 2020 to December 2020 [32]. Participants may have become more accustomed to electronic social communication from spring 2020 to fall 2020, thus enhancing its protective effects.

Furthermore, our results suggest that different forms of online social support matter. In this study only increased online emotional/esteem support was found to be associated with decreased loneliness and depressive symptoms. No other category of online support, including social companionship, had a significant relationship with any outcome variable. These findings suggest that online social support can protect against depressive symptoms aligns with previous research [22], and online emotional/esteem support may be particularly impactful.

### Implications

The present research has significant implications that extend beyond life during a pandemic. First, these findings generally point to the importance of clinicians assessing for level and type of social contact, especially with clients who exhibit symptoms of depression or are at risk for suicide. Second, our findings also indicate the electronic social contact is potentially protective against loneliness, depression, and suicide. Therefore, clinicians should consider helping socially isolated clients to connect with others online, especially for emotional support, such as joining an online support group. This may be especially useful for those individuals who live in rural areas, as they are at increased risk for suicidality [33].

### Limitations

The present study should be interpreted in light of its limitations. First, our study was cross-sectional; therefore, we cannot determine the temporal relationship between COVID-19 related social factors and loneliness, depression, and SI. Future research could employ a longitudinal design to identify the temporal directionality between social factors, depression, and SI. Second, we did not examine suicidal behaviors as an outcome. Some recent research suggests that suicidal behaviors are more related to COVID-19 factors than suicidal thoughts [15], underscoring the importance examining all aspects of suicidality in relation to social distancing. Finally, there are a number of COVID-related factors we did not examine that could impact loneliness, depression, and SI, such as economic hardship resulting from the pandemic. Future research could examine how non-social COVID-related factors interact with social factors to increase risk for loneliness, depressive symptoms, and suicidality.

## Conclusion

Our study indicates that COVID-19 related social contact, particularly electronic social contact, is associated with lower levels of loneliness, SI, and depression. Social distancing practices were not associated with increased loneliness, SI, and depression. Our findings underscore the importance of leveraging electronic methods of social connection, especially among individuals who are at risk for suicide or depression. Clinically, this could involve having pointed discussions with individuals about how to utilize different facets of online social support most effectively during times of crisis to best meet their needs, including emotional ones.

## Supporting information

S1 Dataset (SAV)
